# Bibliometric and visualized analysis of arthroscopic treatment of acromioclavicular joint injury

**DOI:** 10.1186/s13018-023-04193-7

**Published:** 2023-09-26

**Authors:** Jian Zhang, Mingjun Li, Yuxia Yang, Wenkang Liu, Xiangji Meng, Wenyong Fei, Jingcheng Wang

**Affiliations:** 1 Department of Orthopedics, Medical College, Yangzhou University, Yangzhou, China; 2 Department of Orthopedics, Northern Jiangsu People’s Hospital, Dalian Medical University, Dalian, China; 3 Department of Orthopedics, Northern Jiangsu People’s Hospital, Clinical Medical College, Yangzhou University, Yangzhou, China

**Keywords:** Acromioclavicular joint, Coracoclavicular ligament, Arthroscopy, Bibliometric analysis, Visualized analysis

## Abstract

**Background:**

Since arthroscopy was discovered to treat acromioclavicular joint injury, people have had great interest and attention to this beautiful and minimally invasive operation, and related research has been increasing worldwide. At present, there is no bibliometric and visualized analysis in this field. The purpose of this study is to explore the research hotspots and trends of arthroscopic treatment of acromioclavicular joint injury through bibliometric and visualized analysis and look forward to the future development direction of clinical practice.

**Methods:**

The publications on arthroscopic treatment of acromioclavicular joint injury diseases from its establishment to April 2023 were obtained from the Web of Science (WOS) Core Collection database. CiteSpace, VOSviewer, Scimago graphica and Origin were used for bibliometric and visualized analysis.

**Results:**

This study included a total of 330 publications. The number of publications tends to increase every year. The USA has the most significant number of publications and citations. Imhoff AB is the most relevant scholar with the largest number of publications in this field, and the scholar with the highest citation and average citations is Mazzocca AD. Tech Univ Munich, Rush University and Charite are the three institutions with the greatest contribution. Tech Univ Munich, Rush University and Charite are the three institutions with the greatest contribution. In addition, “*Arthroscopy-the Journal of Arthroscopic and Related Surgery”* and “*American Journal of Sports Medicine”* are the institutions with the most significant number of publications and average citations, respectively. The most common keywords are “acromioclavicular joint dislocation,” “arthroscopic resection,” “arthroscopic reconstruction” and “coracoclavicular ligament.”

**Conclusion:**

The number of publications shows a steady upward trend as a whole. However, there is still a lack of cooperation among countries, institutions and scholars around the world, so various countries, institutions and scholars need to strengthen academic exchanges and expand the field of cooperation, so as to promote further research and development in related fields. However, minimally invasive methods such as arthroscopy are still the hotspots and frontiers in the treatment of acromioclavicular joint injury in the future.

## Introduction

Acromioclavicular joint (ACJ) injury is a common shoulder injury in clinic, accounting for about 40% of shoulder injuries [1]. The main types of injuries include ACJ dislocation, acromioclavicular ligament injury, coracoclavicular ligament (vertebral ligament and trapezoid ligament) injury, acromioclavicular arthritis and distal clavicular fracture. In recent years, the incidence of ACJ injury has been increasing yearly, showing a trend of getting younger and younger. Rockwood et al. divided ACJ injury into six types: type I: acromioclavicular (AC) ligament sprain, no ACJ dislocation; type II: AC ligament tear, coracoclavicular ligament intact, ACJ slight dislocation; type III: AC and coracoclavicular (CC) ligament tears, severe ACJ dislocation; type IV: complete dislocation of ACJ with no more than 100% increase in the distance from the coracoid process; type V: complete dislocation of ACJ and the distance from the coracoid process to clavicle increased by 100–300%; and type VI: ACJ inferior dislocation to subacromial or coracoid process [2]. ACJ injury (type III and above) will affect the stability of ACJ, if not timely intervention and treatment, it will seriously affect the activity and function of shoulder joint in the future.

So far, treating patients with ACJ injury is still challenging, and the best management of ACJ injury is still controversial. For patients with mild ACJ injury (type I and type II), satisfactory results can be obtained by conservative treatment such as shoulder and elbow fixation, wearing braces, pain relief and rehabilitation, while patients with severe injuries need surgical treatment (type III–VI). Copper [3] first reported the fixation of ACJ injury in 1986. Since then, more than seventy surgical methods have been reported for ACJ injury [4]. Traditional surgical treatment of ACJ injury mainly includes Kirschner wire technique, hook plate technique, acromioclavicular ligament, coracoclavicular ligament repair and reconstruction, distal clavicle resection and so on [5–8].

In recent years, with the increasing demand for minor trauma and beauty, more and more attention has been paid to minimally invasive treatment of ACJ injury. Since the advent of arthroscopy, it has been used in the treatment of ACJ injury because of its advantages such as less trauma and quick recovery. With the assistance of arthroscopy, suture, thread anchor and clavicular hook plate fixation techniques can be used for anatomical reduction of ACJ dislocation, which is beneficial to restore joint function as soon as possible. Wolf et al. reported for the first time in 2001 that acute ACJ dislocation was treated by arthroscopic coracoclavicular fixation. Polyethylene suture was used for coracoclavicular fixation during the operation [9]. Gomez VLA et al. reported for the first time that ten patients with acute acromioclavicular dislocation were submitted to arthroscopic repair using the TightRope-Arthrex system, and ten cases had a good early functional prognosis [10]. Arthroscopy has gradually become a research hotspot in the field of minimally invasive treatment of ACJ injury.

Bibliometric is a subject that applies mathematical and statistical methods to quantitative information analysis. It can be used to assess the contributions of countries, institutions, journals and authors to specific research topics and to identify research hotspots and trends in a particular field [11]. At present, there is no bibliometric and visualized analysis to summarize the application and development of arthroscopy in ACJ injury diseases. Therefore, the purpose of this paper is to use the research methods of bibliometric to summarize the research in the field of arthroscopic treatment of ACJ injury in the past two decades, and to draw a visualized map by using CiteSpace, VOSviewer, Scimago graphica and Origin to show the hotspots and development trends in this field more intuitively.

## Materials and methods

### Search strategy

The data included in this study are from the Science Citation Index Expanded (SCIE) and Social Sciences Citation Index (SSCI) of the Web of Science (WOS) Core Collection database from its inception to April 30, 2023. In order to ensure the breadth of the search scope, the search terms are constantly filtered, and the final search strategy is as follows: TS = (“acromioclavicular”OR “ligamentum acromioclaviculare”OR “coracoclavicular ligament”OR “ligamentum coracoclaviculare”OR “ligamentum coracoclaviculare”OR “trapezoid ligament”OR “ligamentum trapezoideum”OR “conoid ligament”OR “ligamentum conoideum”OR “distal clavicular fracture”) AND TS = (“Arthroscopy”OR “arthroscope”OR “arthroscopic”). The study included Article, Editorial Material, Letter, Review and Correction related to arthroscopic treatment of ACJ injury. The language of publications is English.

### Data extraction and analysis

After searching SCIE and SSCI in WOSCC database, 392 publications about arthroscopic treatment of ACJ injury were obtained, and some publications not related to arthroscopy or ACJ injury were excluded. Basic information is obtained, including publication types, publication years, publication quantity, countries, institutions, authors, citations, keywords and so on. The publication date is imported into the origin2022 table, and the time trend chart of the annual publication is constructed. We use VOSviewer (1.6.19) to analyze the co-occurrence network of countries, institutions, authors and journals. The size of the nodes in the VOSviewer map indicates the number of publications, the connections between the nodes indicate the intensity of the relationship or collaboration, and the color of each circle indicates the year of publication or cluster [12]. In addition, CiteSpace (6.2.2) also shows the keywords co-occurrence network, cluster diagram, the timeline view of keywords cluster and the strongest keywords of citation bursts. In the map generated by the software, the connection between nodes indicates the correlation of collaboration. Different colors represent different reference years. The software can also analyze the research trends and research hotspots in the specific field of burst keywords recognition [13]. Scimago graphica (1.0.34.0) is used for geospatial visualization.

## Result

### Analysis of publications

**Fig. 1:**
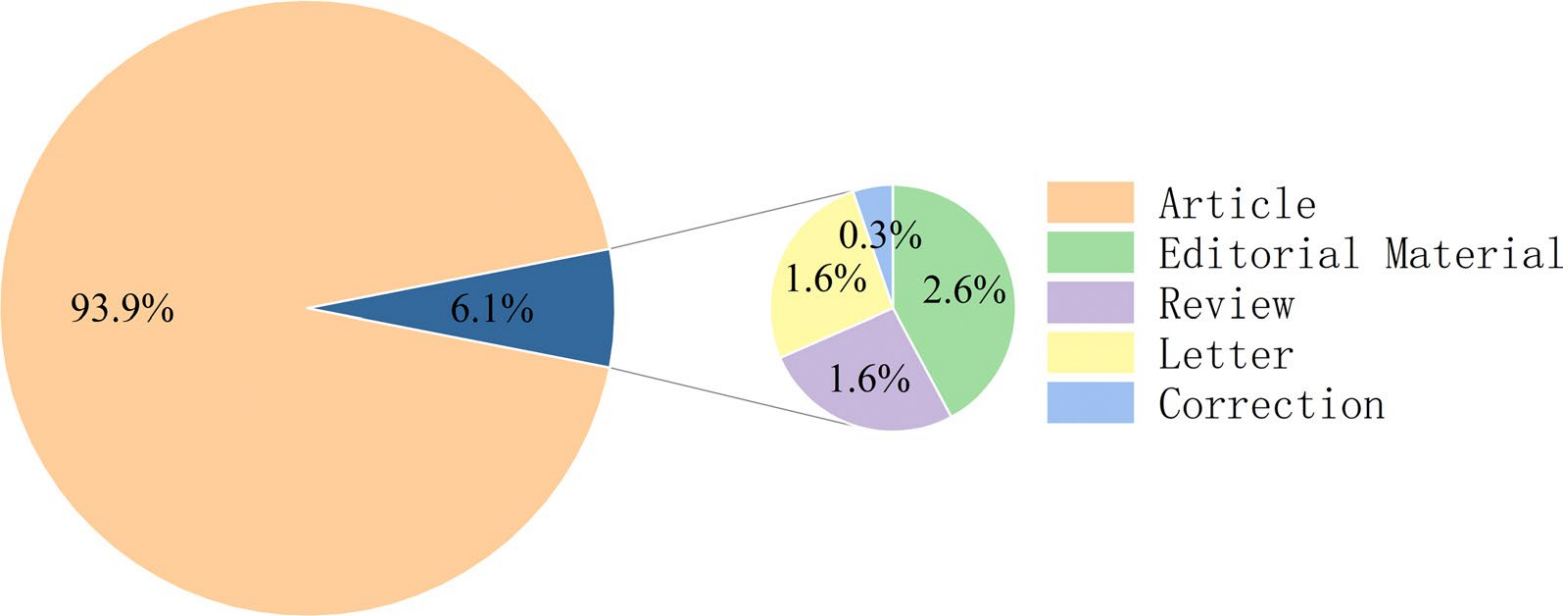
Classification and proportion of included research publications

**Fig. 2:**
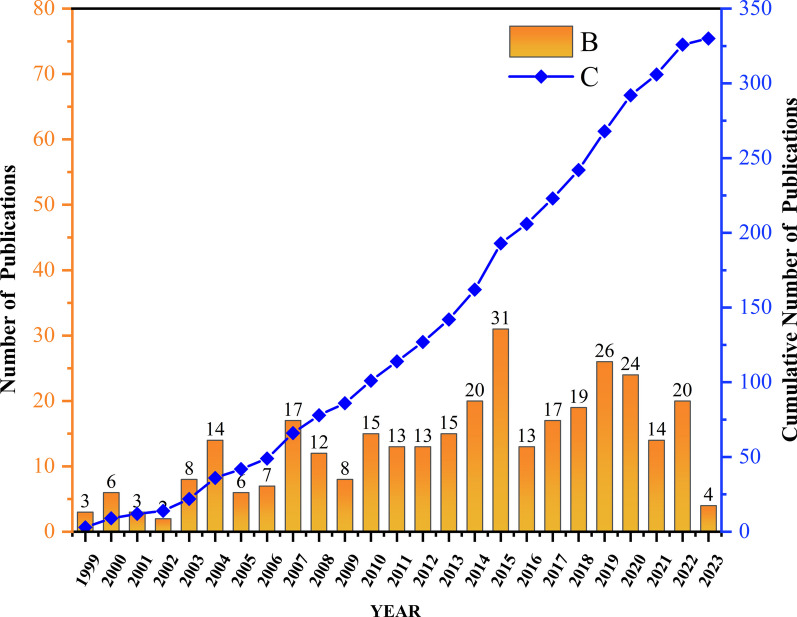
Number of publications from 1999 to 2023 on arthroscopic treatment of ACJ injury

Searches from the WOS database show that from its inception to April 2023, a total of 330 publications finally met the inclusion criteria. As shown in Fig. 1, Article (n = 290, 93.9%), followed by Editorial Material (*n* = 8, 2.6%), Letter (*n* = 5, 1.6%), Review (*n* = 5, 1.6%), and Correction (*n* = 1, 0.3%). The results in Fig. 2 show that the earliest publication on arthroscopic treatment of ACJ injury, published by Matthews LS in 1999, shows that there is no significant difference between arthroscopy and open resection of the distal clavicle [14]. The number of publications showed a significant upward trend. According to the development trend of this research field, the annual publication quantity is divided into three stages. The first stage is from 1999 to 2006. During this period, the number of papers published each year is relatively stable, with 14 published in 2004 and less than 10 in other years. The second stage is from 2008 to 2015. During this period, the number of publications showed a rapid growth trend. The number of annual publications increased from 12 in 2008 to 31 in 2015. More than 1/3 papers were published during this period, with an average annual volume of 15.9. The third stage is from 2016 to 2022, and the number of annual publications showed a slowing growth trend. However, it is worth noting that during this period, the number of annual publications remained high, with an average of about 19 copies per year, the highest in the three stages, indicating an increasing trend in the overall heat of arthroscopic treatment of ACJ injury. The research in related fields is still the focus of the current research. a significant upward trend. According to the development trend of this research field, the annual publication quantity is divided into three stages. The first stage is from 1999 to 2006. During this period, the number of papers published each year is relatively stable, with 14 published in 2004 and less than 10 in other years. The second stage is from 2008 to 2015. During this period, the number of publications showed a rapid growth trend. The number of annual publications increased from 12 in 2008 to 31 in 2015. More than 1/3 papers were published during this period, with an average annual volume of 15.9. The third stage is from 2016 to 2022, and the number of annual publications showed a slowing growth trend. However, it is worth noting that during this period, the number of annual publications remained high, with an average of about 19 copies per year, the highest in the three stages, indicating an increasing trend in the overall heat of arthroscopic treatment of ACJ injury. The research in related fields is still the focus of the current research.

### Analysis of countries and regions

**Fig. 3:**
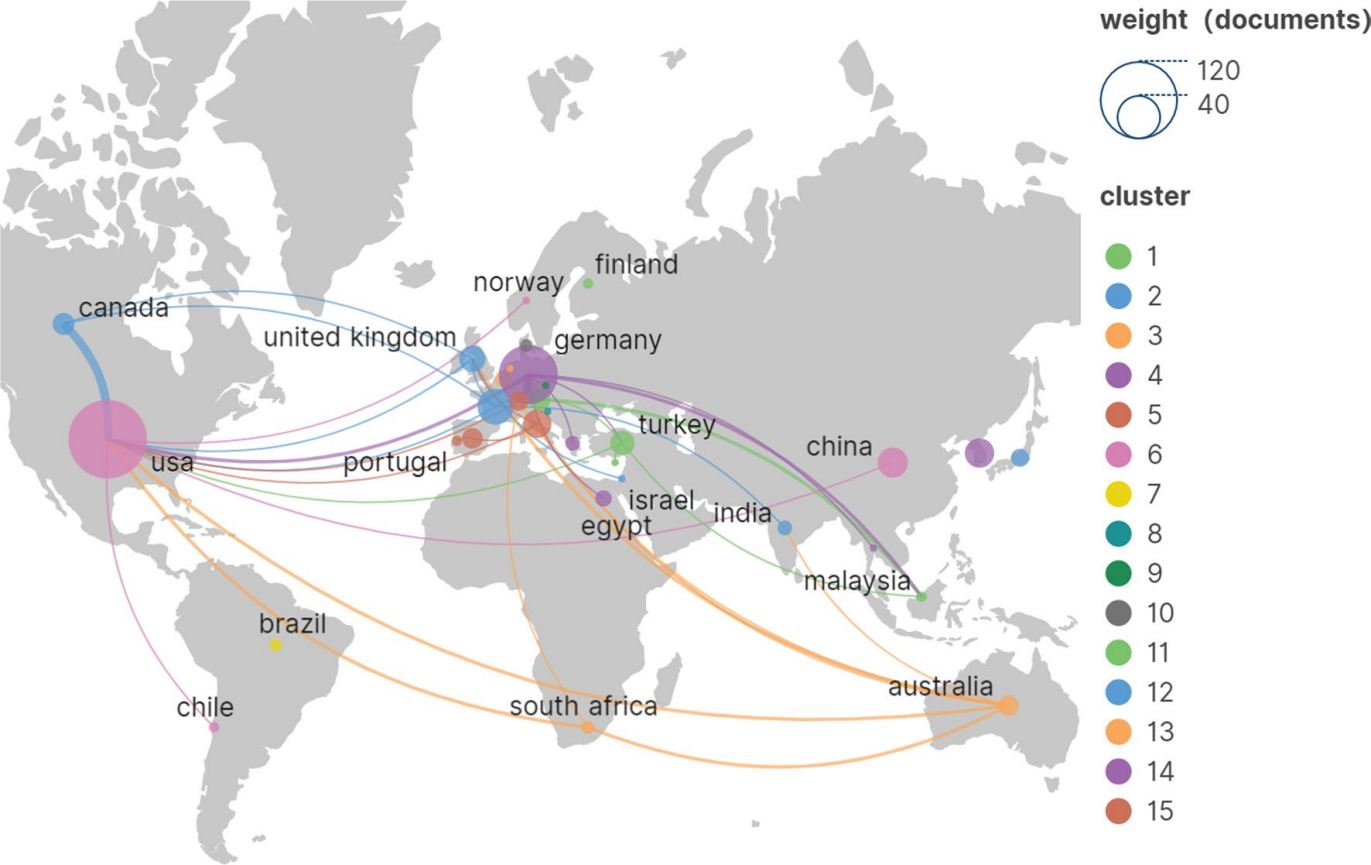
Geographical visualized map of countries on arthroscopic treatment of ACJ injury

**Fig. 4:**
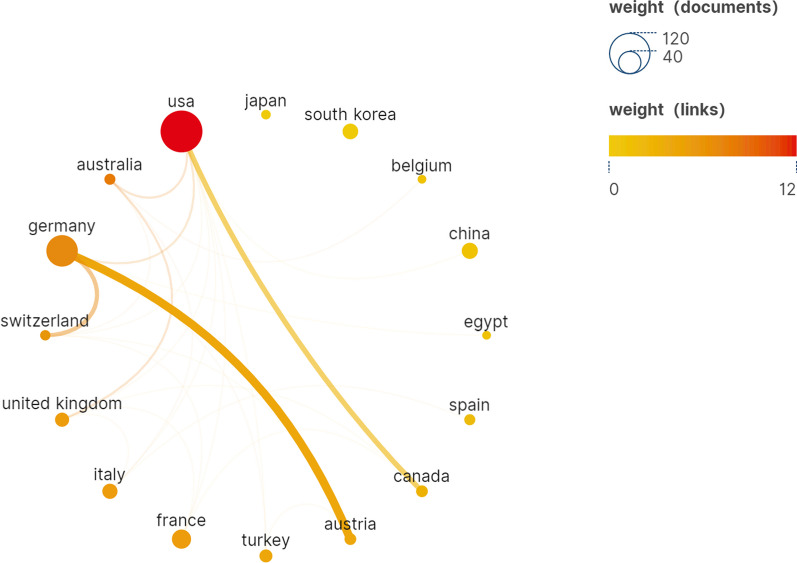
Cooperative network visualized map of countries on arthroscopic treatment of ACJ injury

Geographical maps produced by Scimago graphica show that a total of 35 countries have contributed to the arthroscopic treatment of ACJ injury (Fig. 3). According to the number of publications, Table 1 lists the ten countries with the highest production, of which the USA published the most publications between 1999 and 2023 (*n* = 115, 34.8%), followed by Germany (*n* = 65, 19.7%), France (*n* = 24, 7.3%), China (*n* = 17, 5.2%) and South Korea (*n* = 16, 4.8%). Most of the ten countries with the highest output are concentrated in Europe, the Americas and Asia, of which only Turkey and China are developing countries, and the rest are developed countries. This situation shows that there are obvious regional differences in the study of arthroscopic treatment of ACJ injury, and we think the main reason for the difference is the country’s technological and economic strength. Countries with good technological and economic development may play a leading and core role in related fields. The USA also has the highest citations (*n* = 2,956), about twice as many as the second-ranked UK (*n* = 1467). In terms of the average citations, the USA has the highest average citations (*n* = 25.7). It is worth noting that although only nine papers have been published, the average citations of Austria and Canada are 22.2 and 24.6, respectively, indicating that the quality and research level of publications in these two countries is higher. In the collaboration network generated by Scimago graphica (Fig. 4), the USA and Germany have larger collaboration networks with different countries. Among them, the cooperation between the USA and Canada, Germany and Austria is strong, and there is a strong willingness to cooperate. There is also a small amount of cooperation between other European and American countries, but the Asian countries with a high volume of publications, China and South Korea, have basically no cooperation and exchanges with other countries.

**Table 1:** Top ten countries contributed to publications on arthroscopic treatment of ACJ injury

Rank	Country	Publication	Citations	Average citations
1	USA	115	2956	25.7
2	Germany	65	1467	22.6
3	France	24	544	22.7
4	China	17	123	7.2
5	South Korea	16	213	13.3
6	Italy	15	287	19.1
7	United Kingdom	13	184	14.2
8	Turkey	11	88	8
9	Austria	9	200	22.2
10	Canada	9	221	24.6

### Analysis of institutions

**Fig. 5:**
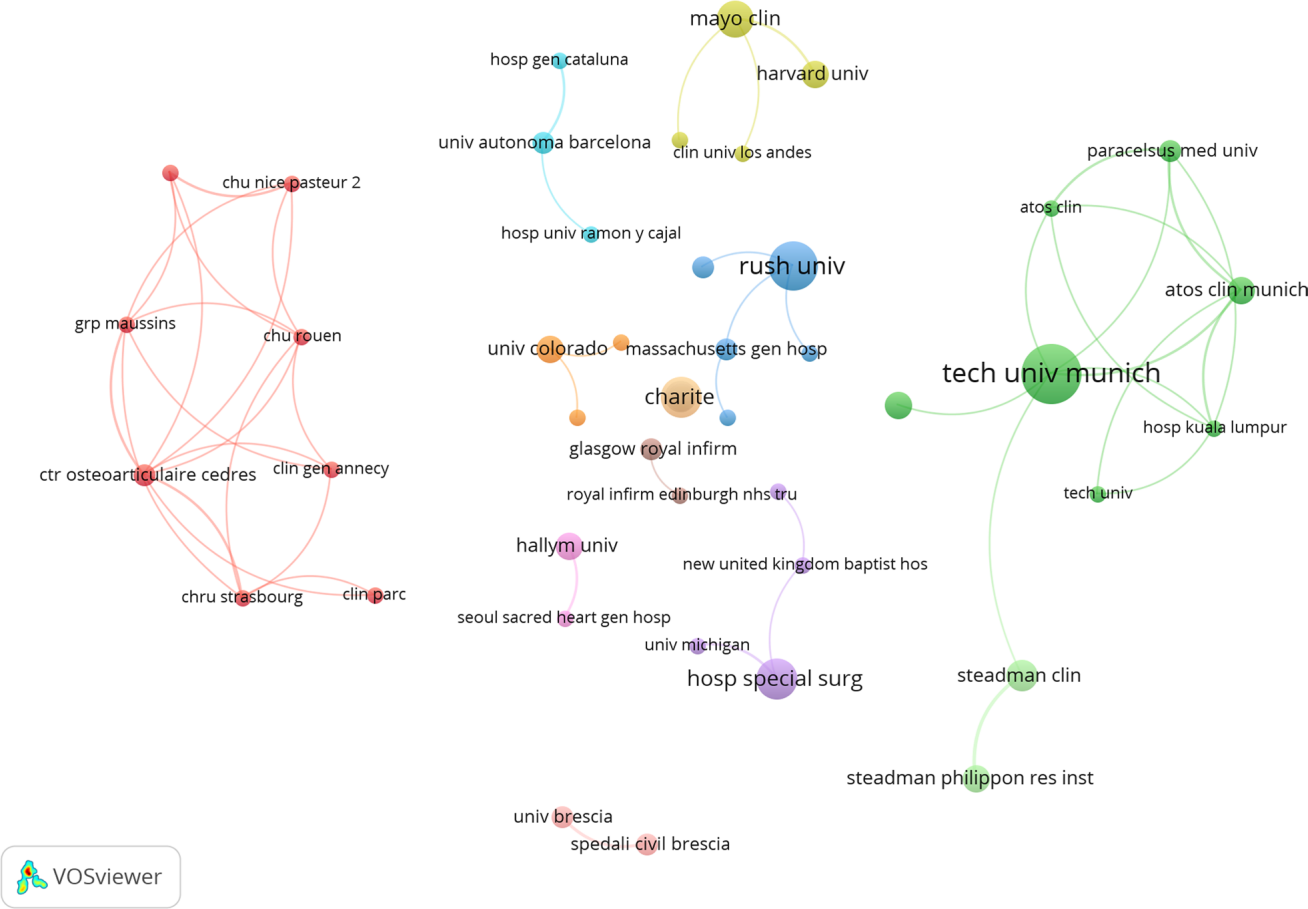
Cooperative network visualized map of institutions on arthroscopic treatment of ACJ injury

A total of 516 institutions have publications on arthroscopic treatment of ACJ injury. As shown in Table 2, the institution with the largest number of publications is Tech Univ Munich (*n* = 12), followed by Rush University (*n* = 9) and Charite (*n* = 7). Among the top ten institutions, Tech Univ Munich has the highest citations (*n* = 349). In addition, it is worth noting that although University of Connecticut has only published five articles, the average citations are the largest (*n* = 146), which is 3.2 times higher than that of the second Charite Univ Med Berlin (*n* = 45.3), indicating that the overall research level of University of Connecticut is high and the quality of the paper is higher. The minimum number of institutional publications is set at 2, and 76 institutions are selected for co-author analysis through the VOSviewer (Fig. 5). 76 institutions form the largest network of institutional co-authors, divided into 45 clusters. The red cluster is the largest, including eight institutions with Ctr Osteoarticulaire Cedres as the center. The green cluster ranks second, focusing on Tech Univ Munich. The top three institutions with the largest total link strength (TLS) are Ctr Osteoarticulaire Cedres (TLS = 9), Rehabilitation and ATOS Clin Munich (TLS = 8) and Tech Univ Munich (TLS = 7). Although these institutions participate in more cooperation than other institutions, the overall cooperation between institutions is still less. Seventy-six institutions form the largest network of institutional co-authors, divided into 45 clusters. The red cluster is the largest, including eight institutions with Ctr Osteoarticulaire Cedres as the center. The green cluster ranks second, focusing on Tech Univ Munich. The top three institutions with the largest total link strength (TLS) are Ctr Osteoarticulaire Cedres (TLS = 9), Rehabilitation and ATOS Clin Munich (TLS = 8) and Tech Univ Munich (TLS = 7). Although these institutions participate in more cooperation than other institutions, the overall cooperation between institutions is still less.

**Table 2:** Top ten institutions contributed to publications on arthroscopic treatment of ACJ injury

Rank	Institution	Publication	Citations	Average citations
1	Tech Univ Munich	12	349	29.1
2	Rush University	9	203	22.6
3	Charite	7	179	25.6
4	Hosp Special Surg	7	169	24.1
5	Mayo Clinic	6	91	15.2
6	University of Connecticut	5	730	146
7	Steadman Clinic	5	91	18.2
8	Charite Univ Med Berlin	4	181	45.3
9	ATOS Clin Munich	4	128	32
10	Harvard University	4	73	18.3

### Analysis of journals

**Fig. 6:**
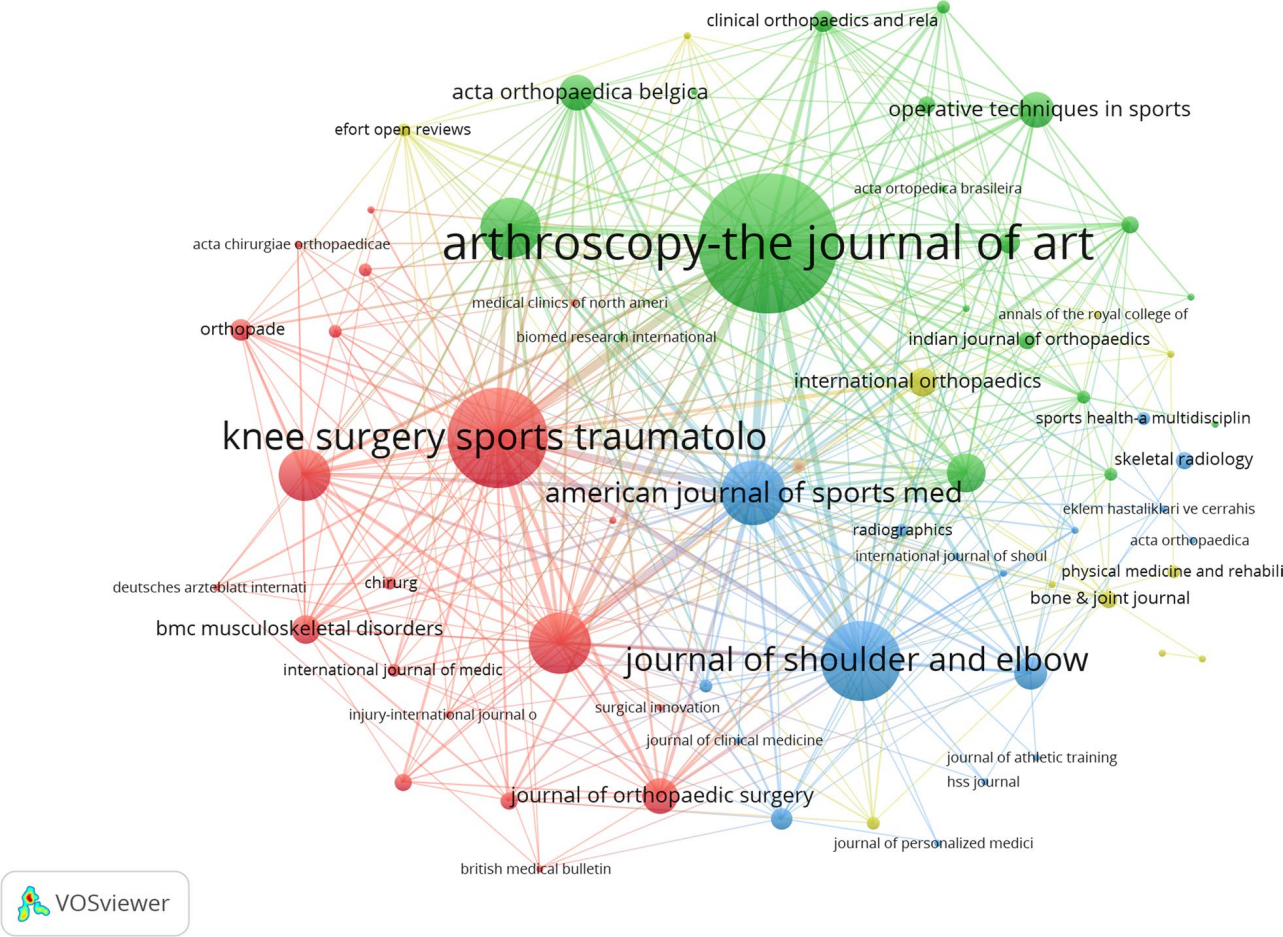
Citation network of journals on arthroscopic treatment of ACJ injury

Between 1999 and 2023, publications related to arthroscopic treatment of ACJ injury were published in 79 journals. Among the ten most published journals listed in Table 3, *Arthroscopy-the Journal of Arthroscopic and Related Surgery* published the most papers (*n* = 52), followed by *Knee Surgery Sports Traumatology Arthroscopy* (*n* = 33) and *Journal of Shoulder and Elbow Surgery* (*n* = 24). Bradford’s Law[15] can be used to identify core journals in different fields, which arranges journals according to the decreasing order of the number of publications, so that the Core Zone with the highest number of publications and the second zone and third zone with the same number of publications as the core can be distinguished in all journals. In this case, the relationship between the number of journals in the core area and the subsequent regions is 1:a:a^2^. Therefore, three core journals are in the current research field (Table 4). They are *Arthroscopy-the Journal of Arthroscopic and Related Surgery*, *Knee Surgery Sports Traumatology Arthroscopy* and d *Journal of Shoulder and Elbow Surgery*, respectively. In terms of citations and average citations, *Arthroscopy-the Journal of Arthroscopic and Related Surgery* has the highest citations (*n* = 1290), followed by *American Journal of Sports Medicine* (*n* = 1206). Among the top ten journals, *American Journal of Sports Medicine* has the highest average citations (*n* = 67), and IF is also the highest (IF = 7.01). VOSviewer is used to create a visualized map of co-citation analysis of journals. The result of Fig. 6 shows a periodical co-citation network with 79 nodes. The node size represents the journals’ status and the number of publications. As shown in Fig. 6, all journals are divided into four clusters. The blue cluster contains journals such as *Journal of Shoulder and Elbow Surgery* and *American Journal of Sports Medicine*, which represent journals related to ACJ injury. The green cluster represented by *Arthroscopy-the Journal of Arthroscopic and Related Surgery* represents the journals related to arthroscopy.

**Table 3:** Top ten journals contributed to publications on arthroscopic treatment of ACJ injury

Rank	Source	Publication	Citations	Average Citations
1	Arthroscopy-the Journal of Arthroscopic and Related Surgery	52	1290	24.8
2	Knee Surgery Sports Traumatology Arthroscopy	33	640	19.4
3	Journal of Shoulder and Elbow Surgery	24	685	28.5
4	American Journal of Sports Medicine	18	1206	67
5	Archives Of Orthopaedic and Trauma surgery	17	398	23.4
6	Orthopaedics & Traumatology-surgery & Research	16	285	17.8
7	Unfallchirurg	13	128	9.8
8	Orthopedics	9	171	19
9	Acta Orthopaedica Belgica	8	104	13
10	Journal Of Orthopaedic Surgery	8	67	8.4

**Table 4:** Classification of core and non-core journals

Zone	Publications/Journal	Number of Journals	Number of Publications
Core zone	≥ 24	3	109
Second zone	4–18	14	128
Third zone	1–3	62	98

### Analysis of authors

**Fig. 7:**
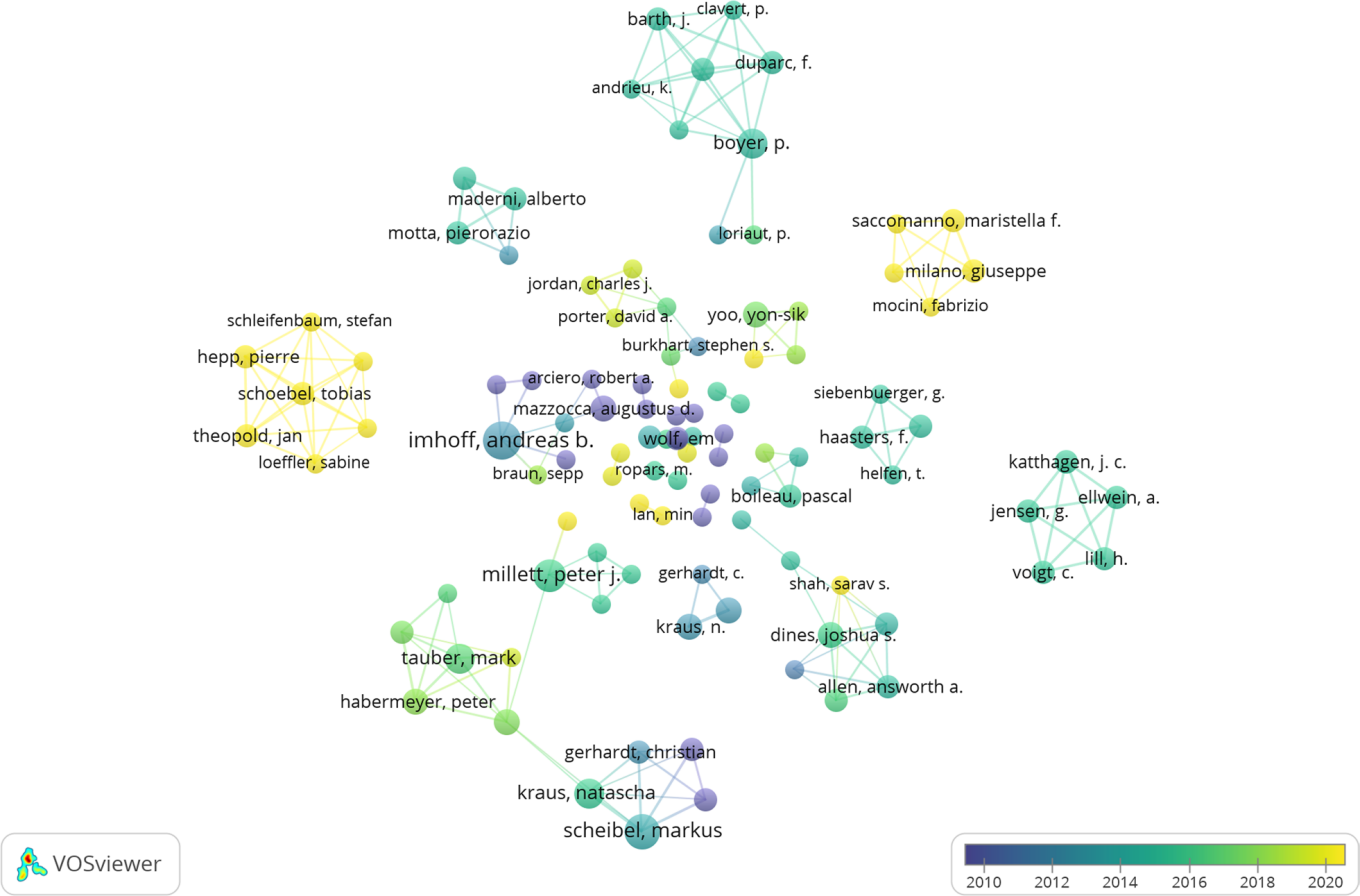
Cooperative network visualized map of authors on arthroscopic treatment of ACJ injury

From 1993 to 2023, 1338 authors published papers related to arthroscopic treatment of ACJ injury. The top ten prolific authors are listed in Table 5. The author with the largest number of publications is Imhoff AB (*n* = 8) [16], followed by Scheibel M (*n* = 7) [17] and Millett PJ (*n* = 6) [18]. It is worth noting that Mazzocca AD[19] with only four publications has the highest citations and average citations, which are 450 and 112.5, respectively. It shows that the research of Mazzocca AD in the field of arthroscopic treatment of ACJ injury is more thorough, the publications are of high quality, and the research is authoritative. According to Price’s law [20], the minimum number of publications by core authors in a certain field is *m* = 0.749x√n_max_, n_max_ represents the number of publications of the most productive authors in the field. According to VOSviewer statistics, *n*_max_ = 8, *m* = 2 is calculated, so authors with more than two articles (including two articles) are designated as the core authors in this field. Among all 1338 authors, there are a total of 117 core authors, accounting for 91% of the total publications. It has reached half of the standards proposed by Price, so it can be considered that a relatively stable cooperative group has been formed in the field of arthroscopic treatment of ACJ injury. In the VOSviewer, we select 117 core authors to make a visualized analysis of the co-author analysis. The network is divided into 38 clusters (Fig. 7). The largest cluster consists of nine authors, but their research is focused on between 2016 and 2018. The Schoebel T[21]-centered cluster (7 authors), whose research in this field is concentrated after 2020, is a relatively new and closely cooperative research team, and it is also a team with great potential in the current field. The top three authors of total link strength are Scheibel [17](TLS = 15), Boyer [22](TLS = 15), and Barth [23] (TLS = 14), which shows that the three authors have done more cooperation with others.

**Table 5:** Top ten institutions contributed to publications on arthroscopic treatment of ACJ injury

Rank	Author	Documents	Citations	Average Citations
1	Imhoff AB	8	360	45
2	Scheibel M	7	315	45
3	Millett PJ	6	92	15.3
4	Kraus N	5	192	38.4
5	Tauber M	5	178	35.6
6	Boyer P	5	146	29.2
7	Mazzocca AD	4	450	112.5
8	Habermeyer P	4	96	24
9	Dines JS	4	92	23
10	Martetschlaeger F	4	69	17.3

### Highly cited publications

Table 6 shows the top ten most cited publications in the field of arthroscopic treatment of ACJ injury. The studies were conducted between 2001 and 2014, with each publication cited at least 87 times. The publication with the highest citation (*n* = 299) was written by Mazzocca AD in 2007 and published in *American Journal of Sports Medicine* entitled “Evaluation and Treatment of Acromioclavicular Joint Injuries.” The intent is to provide a current, in-depth treatise on all aspects of acromioclavicular joint complex injuries to include anatomy, biomechanics, benchmark studies on instability and reconstruction, clinical and radiographic evaluation, and to present the most recent clinical research on surgical outcomes [24]. The second cited publications (*n* = 280) was also written by Mazzocca AD, which shows the core position of Mazzocca AD in this research field.

**Table 6:** Top ten most cited publications on arthroscopic treatment of ACJ injury

Rank	Title	Journal	Author	Year	Citations
1	Evaluation and Treatment of Acromioclavicular Joint Injuries	American Journal of Sports Medicine	Mazzocca, A.D	2007	299
2	A Biomechanical Evaluation of An Anatomical Coracoclavicular Ligament Reconstruction	American Journal of Sports Medicine	Mazzocca, A.D	2006	280
3	All-arthroscopic Versus Mini-open Rotator Cuff Repair: A Long-term Retrospective Outcome Comparison	Arthroscopy-the Journal of Arthroscopic and Related Surgery	Severud, E.L	2003	150
4	Arthroscopic Rotator Cuff Repair With and Without Arthroscopic Subacromial Decompression: A Prospective, Randomized Study of One-year Outcomes	Journal of Shoulder and Elbow Surgery	Gartsman, G.M	2004	114
5	The Geometric classification of Rotator Cuff Tears: a System Linking Tear Pattern to Treatment and Prognosis	Arthroscopy-the Journal of Arthroscopic and Related Surgery	Davidson, J	2010	113
6	Incidence of Associated Injuries with Acute Acromioclavicular Joint Dislocations Types iii through V	American Journal of Sports Medicine	Tischer, T	2009	111
7	The Arthroscopically Assisted Reduction of Acute AC Joint Separations with The Double TightRope Technique Advantages over The Clavicular Hook Plate Fixation?	Knee Surgery Sports Traumatology Arthroscopy	Jensen, G	2014	90
8	The Coracoidal Insertion of The Coracoclavicular Ligaments An Anatomic Study	American Journal of Sports Medicine	Salzmann,GM	2008	89
9	Arthroscopic Reconstruction for Acromioclavicular Joint Dislocation	Arthroscopy-the Journal of Arthroscopic and Related Surgery	Wolf, EM	2001	87
10	Management of Acute Acromioclavicular Joint Dislocations: Current Concepts	Archives of Orthopaedic and Trauma Surgery	tauber, M	2013	83

### Bibliometric and visualized analysis of keywords

**Fig. 8:**
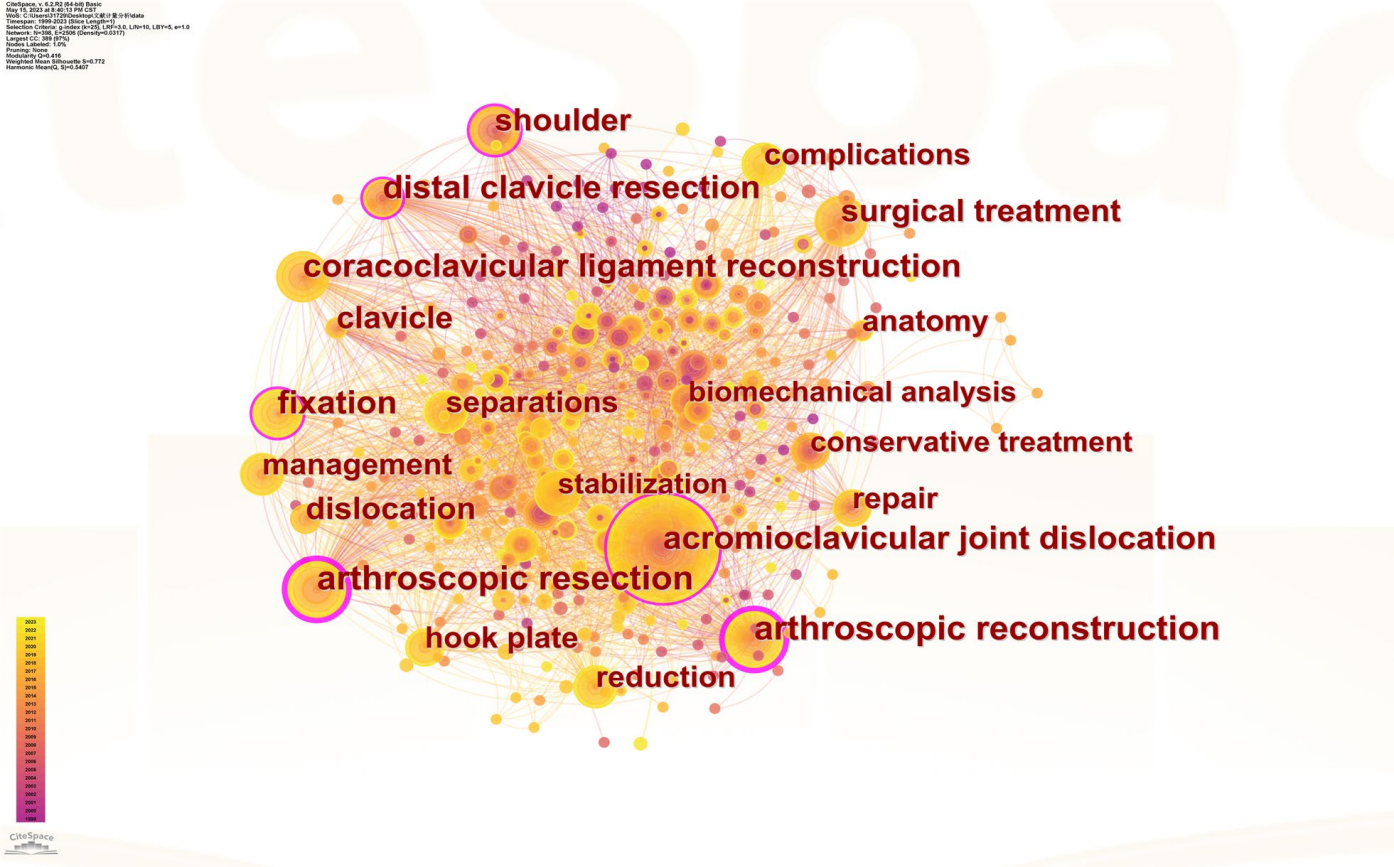
Co-occurrence network map of keywords on arthroscopic treatment of ACJ injury

**Fig. 9:**
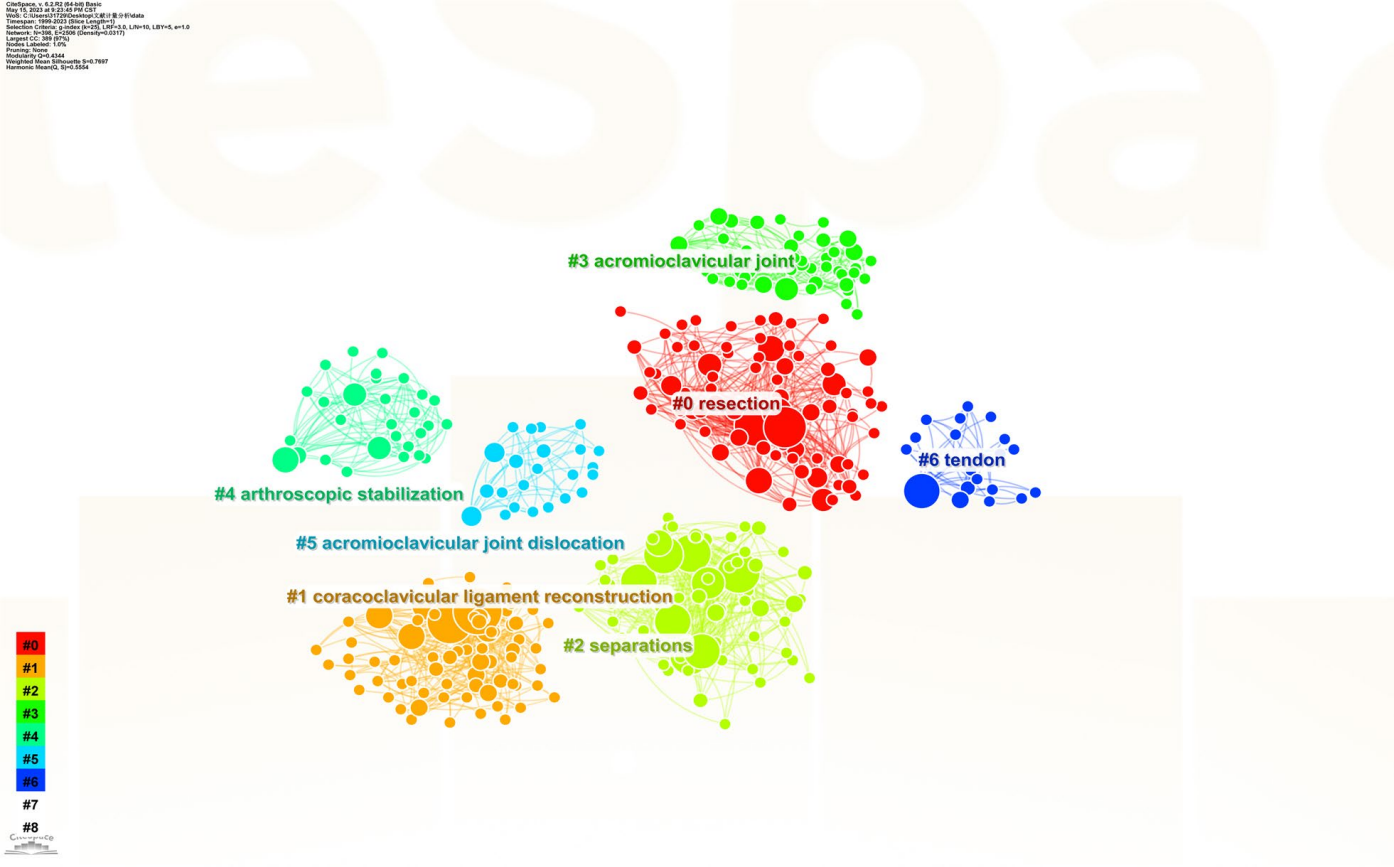
Co-occurrence cluster map of keywords on arthroscopic treatment of ACJ injury

**Fig. 10:**
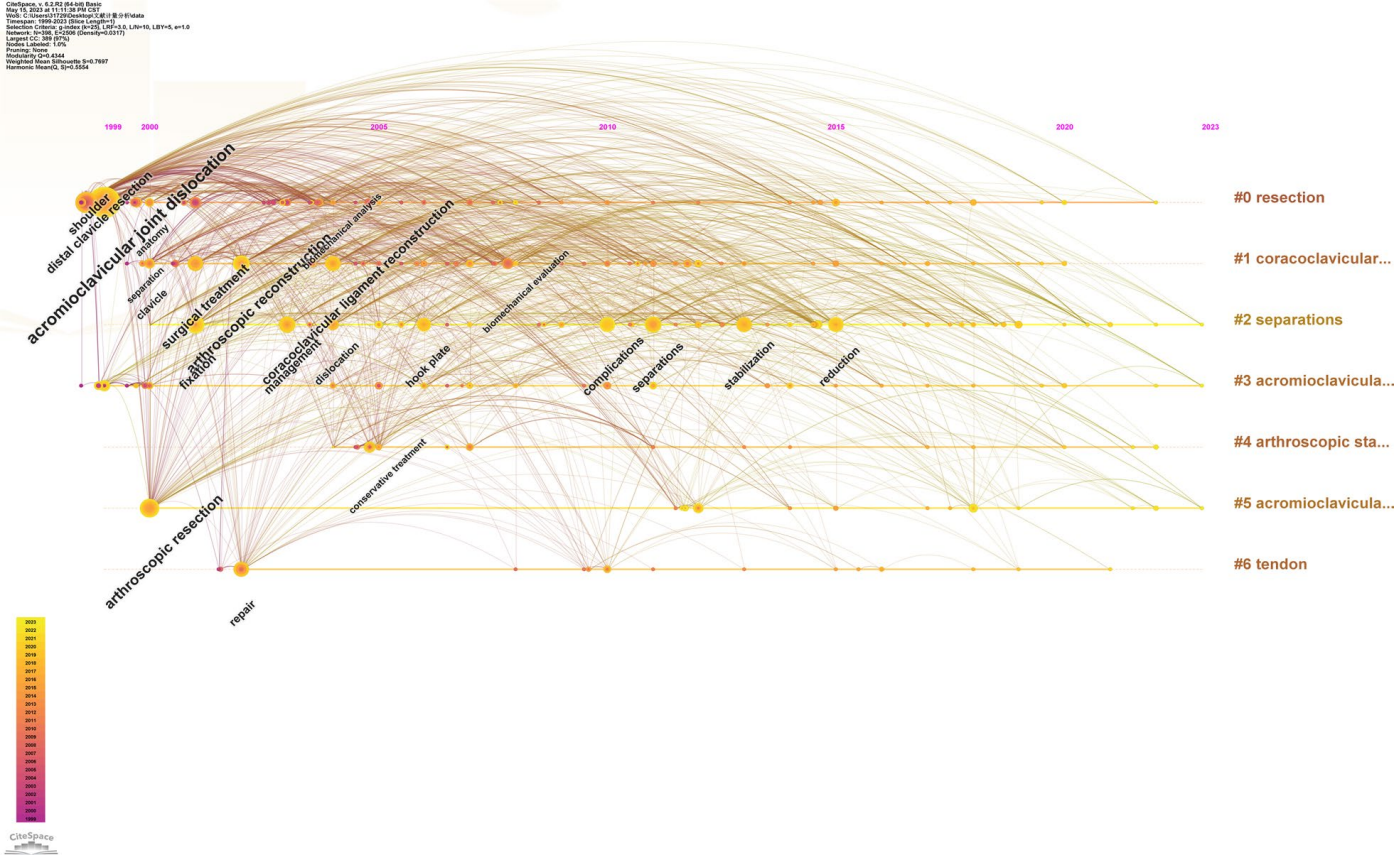
Keywords clustering sequence map on arthroscopic treatment of ACJ injury

**Fig. 11:**
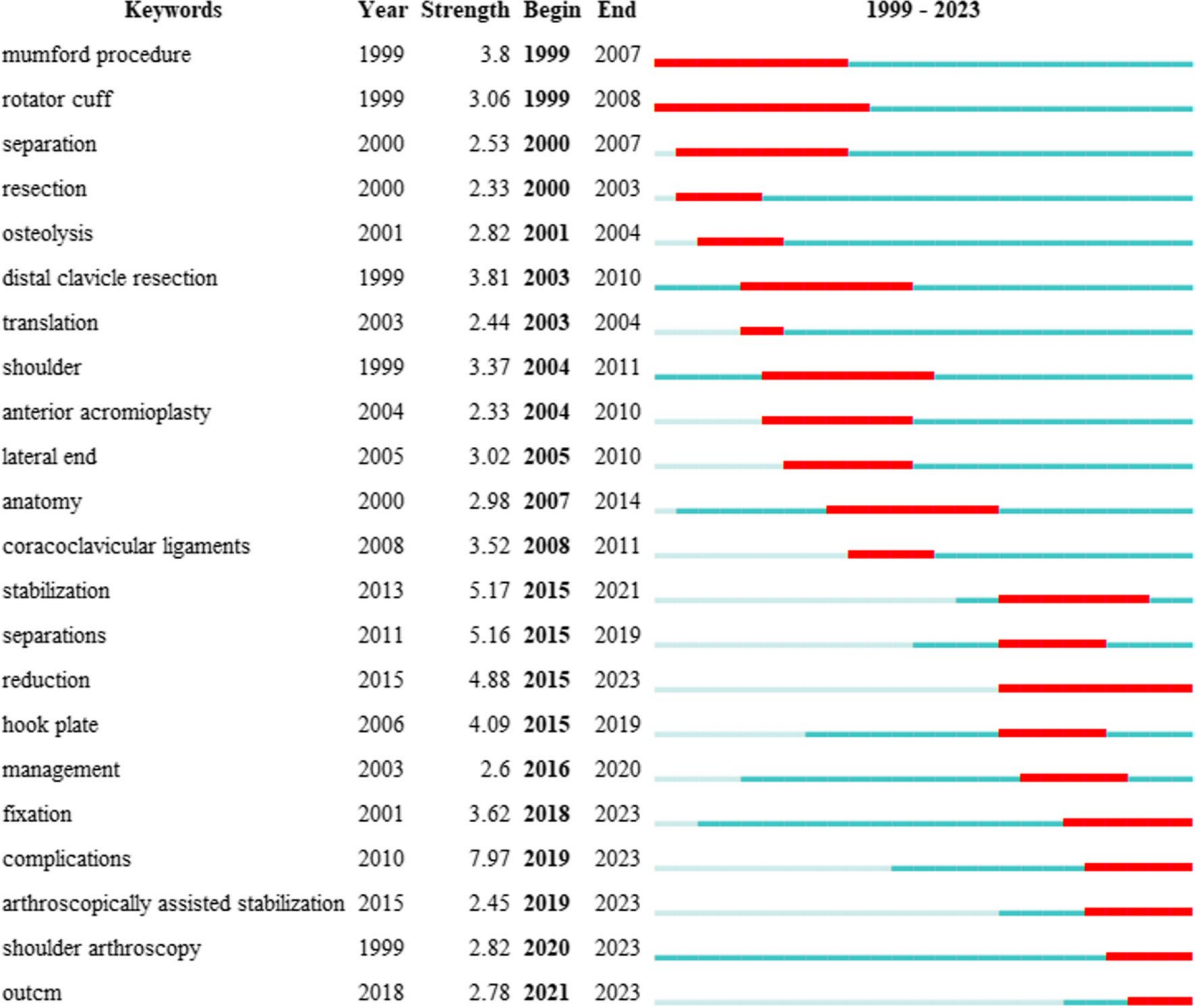
Top 22 keywords with the strongest citation bursts

Using CiteSpace to analyze the keywords, a co-occurrence network with 398 nodes and 2506 connections is obtained. After merging similar words, a keyword co-occurrence graph is obtained (Fig. 8). The top 20 keywords with the highest frequency are shown in Table 7, and the top five most frequently used keywords are “acromioclavicular joint dislocation” (158 times), “arthroscopic resection” (72 times), “arthroscopic reconstruction” (70 times), “coracoclavicular ligament” (47 times) and “surgical treatment” (44 times). The size of the centrality of keywords should not only study the importance and influence, but also reflect the research hotspots in the field. However, not all high-frequency keywords have high centrality at the same time. The key words with high centrality are “arthroscopic resection” (centrality = 0.23), “arthroscopic reconstruction” (centrality = 0.21), “acromioclavicular joint dislocation” (centrality = 0.19), “distal clavicle resection” (centrality = 0.15) and “fixation” (centrality = 0.11). After clustering the keywords, seven clusters are generated (Fig. 9). The Q value is 0.4344, which indicates that the clustering structure is significant, and the S value is 0.7697, which indicates that the clustering structure is reasonable. In Fig. 9, seven clusters are divided into two categories according to the types of keywords in each cluster. The high-frequency keywords included in the first category of clustering are #5 “acromioclavicular joint dislocation,” #3 “acromioclavicular joint,” #2 “separation” and 6# “tendon.” It mainly describes which diseases or injuries belong to ADJ injury. The second category of clustering includes 4# “arthroscopic stabilization,”1# “coracoclavicular ligament reconstruction” and 0# “resection,” which represents different types of ACJ injury using various arthroscopic surgery methods. Figure 10 is the keywords sequence diagram of arthroscopic treatment of ACJ injury, which can reflect different development processes and interrelationships. As shown in Fig. 10, the evolution process of 0# “resection” is the most abundant and has the deepest influence on each cluster. In addition, we generated a map with the strongest reference to the burst keywords. Figure 11 lists the top 22 keywords. The five keywords with the highest bursting power are “complication,” “stabilization,” “separation,” “reduction” and “hook plate.” It shows that these words have a high burst rate in a short time, which is a hot topic in this field. In addition, the keywords “fixation,” “complications,” “arthroscopically assisted stabilization” and “shoulder arthroscopy” have also surged in recent years, indicating the hotspots and frontier of research in this field.

**Table 7:** Top 20 most frequently used keywords

Number	Keyword	Court	Centrality	Year
1	acromioclavicular joint dislocation	158	0.19	1999
2	arthroscopic resection	72	0.23	2000
3	arthroscopic reconstruction	70	0.21	2002
4	coracoclavicular ligament reconstruction	47	0.09	2004
5	surgical treatment	44	0.09	2001
6	distal clavicle resection	38	0.15	1999
7	shoulder	36	0.10	1999
8	fixation	34	0.11	2001
9	complications	31	0.05	2010
10	management	30	0.06	2003
11	separations	28	0.05	2011
12	hook plate	27	0.04	2006
13	stabilization	26	0.06	2013
14	reduction	26	0.06	2015
15	repair	25	0.09	2002
16	dislocation	22	0.06	2004
17	clavicle	19	0.08	2000
18	anatomy	18	0.08	2000
19	conservative treatment	14	0.05	2005
20	biomechanical analysis	13	0.03	2004

## Discussion

The purpose of this study is to use bibliometric research methods to summarize the research in the field of arthroscopic treatment of ACJ injury in the past 20 years and show its research hotspots and frontiers. A total of 330 studies related to this field were included from WOS. As shown in Fig. 2, the annual publication volume is mainly divided into three stages: the low-yield period before 2006, probably because the research field has just started during this period, and the arthroscopic treatment of ACJ injury disease is still in the exploratory stage. The research is mainly focused on arthroscopic exploration [25], subacromial decompression [26] and distal clavicle resection [14, 27]. With the development of arthroscopic technology and the improvement of heat, the field developed rapidly from 2007 to 2015. The main research hotspots during this period are arthroscopic distal clavicle resection [28] and arthroscopic reconstruction of acromioclavicular ligament and coracoclavicular ligament [17, 29, 30]. The development speed has slowed since 2015, but it has maintained a steady growth. Although the focus of this period is still focused on arthroscopic reconstruction of acromioclavicular ligament and coracoclavicular ligament in the treatment of acute ACJ dislocation, ligament reconstruction techniques have been improved [31–35]. The USA and Tech Univ Munich are the leading countries and institutions in this field, with 115 and 12 publications, respectively. As shown in Table 1 and Fig. 3, the USA, Germany, France, China and South Korea have a relatively high volume of publications, and the relevant papers are mainly published in Europe and America and the regions with better economic development in Asia. It can be seen that the development of research is closely related to technological and economic development, and countries with good technological and economic development play a leading role in this field. It is worth noting that there is very little cooperation and exchanges between different countries, and Fig. 4 shows that there are exchanges and cooperation in a small number of European and American countries, while China and South Korea, which have a high volume of publications in Asia, basically do not cooperate with other countries. Institutions in the same country have formed a relatively stable network of co-authors, but institutions in different countries have less cooperation. Table 5 shows that Imhoff [16] is the most prolific author (8 publications). Mazzocca [24] is the most cited author (450 times) and the most highly cited author (112.5 times) on average, and is also the most influential person in the field. There are 117 core authors in this field, and each author has formed a stable cooperation team, but there is still a lack of cooperation and communication among the teams. In the future, it is very necessary to treat ACJ injury based on arthroscopy all over the world. Therefore, it is suggested that researchers from various countries should strengthen technical exchanges and cooperation with researchers from other countries on the basis of their own research and produce more and higher-quality research all over the world, so as to promote the further development of this research field.

For the visualized analysis of journals, according to the results of Tables 3 and 4, the top three high-yield journals are *Arthroscopy-the Journal of Arthroscopic and Related Surgery*, *Knee Surgery Sports Traumatology Arthroscopy and Journal of Shoulder and Elbow Surgery.* The total number of publications in the first three journals is 109, accounting for about 1/3 of the total number of papers published in this field, indicating that these three journals are the core journals in this field. And the JCR divisions of these three journals all belong to Q1 and Q2, which means that the quality of these journals is relatively high. With the rapid development of arthroscopic technology, perhaps more papers will be published in these journals in the future. Table 6 shows that “Evaluation and Treatment of Acromioclavicular Joint Injuries” and “A Biomechanical Evaluation of An Anatomical Coracoclavicular Ligament*”* are the two most cited articles in this field [24, 36]. Relevant researchers can also pay attention to the above journals and articles.

As can be seen from the keywords co-occurrence diagram (Fig. 8), the keywords with the highest frequency are** “**acromioclavicular joint dislocation,” “arthroscopic resection,” “arthroscopic reconstruction” and “coracoclavicular ligament.” It shows that the most important research in the field of arthroscopic treatment of ACJ injury is arthroscopic coracoclavicular ligament reconstruction and arthroscopic resection of the distal clavicle. According to the different types of keywords in each group, the keywords are divided into seven clusters (Fig. 9), and the seven clusters are divided into two categories. The first category mainly includes ACJ-related diseases, including ACJ dislocation and ACJ ligament and tendon injury. The second category mainly uses arthroscopy to treat ACJ-related diseases. The keywords burst map indicates that the words “fixation,” “complications,” “arthroscopically assisted stabilization” and “shoulder arthroscopy” have surged in recent years, which may be the frontiers and hotspots of current and future research. Researchers in this field can focus on these keywords.

At present, with the continuous development of arthroscopic technology, more and more doctors try to use arthroscopic technology to treat ACJ injury. The focus of research is from initial arthroscopic exploration, subacromial decompression under arthroscopy to arthroscopic distal clavicle resection, arthroscopic ligament reconstruction and various improved techniques, so as to achieve the effect of less trauma and rapid recovery.

## Advantages and limitation

We used CiteSpace, VOSviewer, Scimago graphica and Origin to make a bibliometric and visualized analysis of the annual number of publications, countries, institutions, journals, authors and keywords of arthroscopic treatment of ACJ injury. Although this study is the first bibliometric study based on arthroscopy in the treatment of ACJ injury, it also has some limitations. First of all, although WOS is an authoritative database containing various publications suitable for the subject of this study, one database cannot contain all the research. Secondly, only English publications are retrieved and included in this study, so studies in other languages may be ignored. As a result, the number of search results may be different from the actual number of posts. Therefore, we put forward some suggestions for future research. In future research, the extraction of publications can include more databases such as PubMed and Medline. At the same time, publications in other languages can also be included, so as to increase the reliability of the research.

## Conclusion

Based on bibliometric analysis, this study has shown a steady increase in the total amount of publication since 1999. The USA, Germany, France, China and South Korea have played a leading role in the arthroscopic treatment of acromioclavicular joint injuries, but there is still a relative lack of cooperation among countries around the world. The focus of research is still focused on arthroscopic resection of the distal clavicle and ligament repair and reconstruction. What relevant researchers need to do is to pay attention to the core journals, influential authors, highly cited articles and recent research hotspots and trends in this field, and strengthen exchanges and cooperation among countries, regions, institutions and authors in order to make the development of this field further.regions, institutions and authors in order to make the development of this field further.

## Data Availability

The data behind this article will be shared with the appropriate authors with reasonable requirements.

## References

[1] Koch M, Werner A, Engel G, Huth J, Mauch F (2023). Mini-open vs. arthroscopic double tight-rope reconstruction after acute AC-joint dislocation: a comparison in functional outcome and sports activity. Arch Orthop Trauma Surg.

[2] Gorbaty JD, Hsu JE, Gee AO (2017). Classifications in brief: rockwood classification of acromioclavicular joint separations. Clin Orthop Relat Res.

[3] Cooper ES (1970). Art. VI.-New Method of treating long standing dislocations of the Scapulo-clavicular articulation. Am J Med Sci.

[4] Berthold DP, Muench LN, Dyrna F, Mazzocca AD, Garvin P, Voss A, Scheiderer B, Siebenlist S, Imhoff AB, Beitzel K (2022). Current concepts in acromioclavicular joint (AC) instability–a proposed treatment algorithm for acute and chronic AC-joint surgery. BMC Musculoskelet Disord.

[5] Gumina S, Carbone S, Postacchini F (2009). Scapular dyskinesis and sick scapula syndrome in patients with chronic type III acromioclavicular dislocation. Arthrosc J Arthrosc Relat Surg.

[6] Murena L, Canton G, Vulcano E, Cherubino P (2013). Scapular dyskinesis and SICK scapula syndrome following surgical treatment of type III acute acromioclavicular dislocations. Knee Surg Sports Traumatol Arthrosc.

[7] Pallis M, Cameron KL, Svoboda SJ, Owens BD (2012). Epidemiology of acromioclavicular joint injury in young athletes. Am J Sports Med.

[8] Schlegel TF, Burks RT, Marcus RL, Dunn HK (2001). A prospective evaluation of untreated acute grade III acromioclavicular separations. Am J Sports Med.

[9] Wolf EM, Pennington WT (2001). Arthroscopic reconstruction for acromioclavicular joint dislocation. Arthrosc J Arthrosc Relat Surg.

[10] Gomez Vieira LA, Visco A, DaneuFernandes LF, Gomez Cordero NG (2009). Arthroscopic treatment of acromioclavicular joint dislocation by tight rope technique (Arthrex()). Revista brasileira de ortopedia.

[11] Smith DR (2008). Bibliometrics, dermatology and contact dermatitis. Contact Dermat.

[12] van Eck NJ, Waltman L (2017). Citation-based clustering of publications using CitNetExplorer and VOSviewer. Scientometrics.

[13] Kastrin A, Hristovski D (2021). Scientometric analysis and knowledge mapping of literature-based discovery (1986–2020). Scientometrics.

[14] Matthews LS, Parks BG, Pavlovich LJ, Giudice MA (1999). Arthroscopic versus open distal clavicle resection: a biomechanical analysis on a cadaveric model. Arthroscopy.

[15] Donthu N, Kumar S, Mukherjee D, Pandey N, Lim WM (2021). How to conduct a bibliometric analysis: an overview and guidelines. J Bus Res.

[16] Imhoff AB (2014). Shoulder Girdle, AC and SC-Joints. Oper Orthop Und Traumatol.

[17] Scheibel M, Ifesanya A, Pauly S, Haas NP (2008). Arthroscopically assisted coracoclavicular ligament reconstruction for chronic acromioclavicular joint instability. Arch Orthop Trauma Surg.

[18] Millett PJ, Braun S, Gobezie R, Pacheco IH (2009). Acromioclavicular joint reconstruction with coracoacromial ligament transfer using the docking technique. BMC Musculoskelet Disord.

[19] Mazzocca AD, Conway JE, Johnson S, Rios CG, Dumonski ML, Santangelo SA, Arciero RA (2004). The anatomic coracoclavicular ligament reconstruction. Oper Tech Sports Med.

[20] Linnenluecke MK, Marrone M, Singh AK (2020). Conducting systematic literature reviews and bibliometric analyses. Aust J Manag.

[21] Schoebel T, Theopold J, Fischer J-P, Loeffler S, Schleifenbaum S, Hepp P (2022). Anatomical versus non-anatomical configuration of double coraco-clavicular tunnel technique in acromioclavicular joint reconstruction. Arch Orthop Trauma Surg.

[22] Issa SP, Payan C, Le Hanneur M, Loriaut P, Boyer P (2018). Arthroscopically assisted reduction of acute acromioclavicular joint dislocation using a single double-button device: medium-term clinical and radiological outcomes. Orthop Traumato Surg Res.

[23] Barth J, Duparc F, Andrieu K, Duport M, Toussaint B, Bertiaux S, Clavert P, Gastaud O, Brassart N, Beaudouin E, De Mourgues P, Berne D, Bahurel J, Najihi N, Boyer P, Faivre B, Meyer A, Nourissat G, Poulain S, Bruchou F, Menard JF (2015). Is coracoclavicular stabilisation alone sufficient for the endoscopic treatment of severe acromioclavicular joint dislocation (Rockwood types III, IV, and V)?. Orthop Traumatol Surg Res.

[24] Mazzocca AD, Arciero RA, Bicos J (2007). Evaluation and treatment of acromioclavicular joint injuries. Am J Sports Med.

[25] Mohtadi NG, Vellet AD, Clark ML, Hollinshead RM, Sasyniuk TM, Fick GH, Burton PJ (2004). A prospective, double-blind comparison of magnetic resonance imaging and arthroscopy in the evaluation of patients presenting with shoulder pain. J Shoulder Elbow Surg.

[26] Fischer BW, Gross RM, McCarthy JA, Arroyo JS (1999). Incidence of acromioclavicular joint complications after arthroscopic subacromial decompression. Arthrosc J Arthrosc Relat Surg.

[27] Sellards R, Nicholson GP (2004). Arthroscopic distal clavicle resection. Oper Tech Sports Med.

[28] Hardeman F, Van Rooyen K, Somers J, Doll S, Page R, De Beer J (2013). Biomechanical comparison of indirect and direct arthroscopic excision of the distal clavicle. Acta Orthop Belg.

[29] Kany J, Amaravathi RS, Guinand R, Valenti P (2012). Arthroscopic acromioclavicular joint reconstruction using a synthetic ligament device. Eur J Orthop Surg Traumatol.

[30] Pan Z, Zhang H, Sun C, Qu L, Cui Y (2015). Arthroscopy-assisted reconstruction of coracoclavicular ligament by Endobutton fixation for treatment of acromioclavicular joint dislocation. Arch Orthop Trauma Surg.

[31] Boileau P, Gastaud O, Wilson A, Trojani C, Bronsard N (2019). All-arthroscopic reconstruction of severe chronic acromioclavicular joint dislocations. Arthrosc J Arthrosc Relat Surg.

[32] Campagna V, Piccinni V, Rotundo G, Candela V, Gumina S (2021). The Kite technique: a new all-arthroscopic technique for the treatment of acute acromioclavicular joint dislocation. Knee Surg Sports Traumatol Arthrosc.

[33] Dias CM, Leite MJ, da Silva MR, Granate P, Teixeira JM (2022). Arthroscopic anatomical acromioclavicular joint reconstruction using a button device and a semitendinosus graft. Orthop Surg.

[34] Jiang H, Tong J, Shen L, Jin G, Zhu R (2022). Clinical outcomes of arthroscopy-assisted modified triple endobutton plate fixation in rockwood type III acute acromioclavicular joint dislocation: a retrospective study. Orthop Surg.

[35] Xu J, Liu H, Lu W, Li D, Zhu W, Ouyang K, Wu B, Peng L, Wang D (2018). A retrospective comparative study of arthroscopic fixation in acute Rockwood type IV acromioclavicular joint dislocation: single versus double paired Endobutton technique. BMC Musculoskelet Disord.

[36] Mazzocca AD, Santangelo SA, Johnson ST, Rios CG, Dumonski ML, Arciero RA (2006). A biomechanical evaluation of an anatomical coracoclavicular ligament reconstruction. Am J Sports Med.

